# The epicondylar ratio can be reliably determined in both computed tomography and X-ray

**DOI:** 10.1007/s00402-021-03888-y

**Published:** 2021-04-11

**Authors:** Bernd Lutz, Lucia Polcikova, Martin Faschingbauer, Heiko Reichel, Ralf Bieger

**Affiliations:** Department of Orthopaedic Surgery, University of Ulm, Ober Eselsberg 45, 89081 Ulm, Germany

**Keywords:** Revision total knee arthroplasty, Joint line, Knee replacement, Epicondylar ratio

## Abstract

**Purpose:**

One of the key factors to the successful revision of total knee arthroplasty (rTKA) is the reconstruction of the joint line, which can be determined using the epicondylar ratio (ER). The measurement is established in X-ray and MRI. However, it is not known whether computed tomography (CT) allows a more reliable determination. The objective was to assess the reliability of the ER in CT and to determine the correlation between the ER in CT and a.p. X-ray of the knee.

**Methods:**

The ER was determined on X-ray and CT images of a consecutive series of 107 patients, who underwent rTKA. Measurements were made by two blinded observes, one measured twice. The inter- and intraobserver agreement, as well as the correlation between the two methods, were quantified with the Intraclass Correlation Coefficient.

**Results:**

The average lateral ER was 0.32 (± 0.04) in X-ray and 0.32 (± 0.04) in CT. On the medial side, the average ER was 0.34 (± 0.04) in X-ray and 0.35 (± 0.04) in CT. The interobserver agreement for the same imaging modality was lateral 0.81 and medial 0.81 in X-ray as well as lateral 0.74 and medial 0.85 in CT. The correlation between the two methods was lateral 0.81 and medial 0.79.

**Conclusions:**

The ER can be reliably determined in X-ray and CT. Measurements of the two image modalities correlate. Prior to rTKA, the sole use of the X-ray is possible.

## Introduction

In revision total knee arthroplasty (rTKA), the surgeon is often confronted with femoral or tibial bone loss, which leads to difficulties in reconstructing the joint line (JL) [3]. An anatomical JL position has shown better clinical results and a better outcome compared to a more proximal or distal JL position [3]. JL elevation results in impingement of the inferior patellar pole or the patellar tendon against the inlay, which causes structural damage of the patellar tendon and might lead to failure of the extensor mechanism [12]. Additionally, an elevated JL increases the patellofemoral compression forces and alters knee kinematics [6, 19]. Higher patellofemoral compression forces are associated with anterior knee pain, decreased range of motion, increased polyethylene wear, and a decreased implant survival rate [6, 11, 12]. The restoration of the JL within a threshold of ± 5 mm is considered to be acceptable [10, 14, 18].

There are multiple techniques to estimate the JL position [2, 4, 9, 15]. Currently, relative methods like the adductor tubercle and the epicondylar ratio (ER) are used additionally. In a retrospective study, Bieger et al. correlated the ER and the Figgie distance with the clinical outcome [1]. In their series of TKA revisions, the ER correlated better with the clinical outcome. However, the ideal imaging modality for measuring the ER is not clear. The ER was originally described on magnetic resonance imaging (MRI) [15], but in the case of revision TKA (rTKA) metallic artifacts make measurements adjacent to implants challenging [17]. A correlation between the ER measured on MRI and X-ray of the knee as well as a reliable measurement technique in X-ray has been demonstrated [8].

The use of computed tomography (CT) for measuring the ER prior to rTKA has not been evaluated. Since the distance from the epicondyle to the JL heavily influences the ER we evaluated the CT for an alternative. We hypothesized that determining the ER in CT would be more reliable than X-ray of the knee.

## Materials and methods

In a retrospective study, a consecutive series of 107 patients, who underwent rTKA between 2014 and 2017 were analyzed. The inclusion criteria were CT and X-ray in acceptable quality, at best same-day CT and X-ray, and no severe fracture or component dislocation. The included 42 female and 65 male patients had either the diagnosis of aseptic loosening, component malposition, instability, arthrofibrosis, anterior knee pain, periprosthetic fracture or periprosthetic joint infection. The study was approved by the local ethical committee (application 243/17).

### Definition of landmarks

**Fig. 1:**
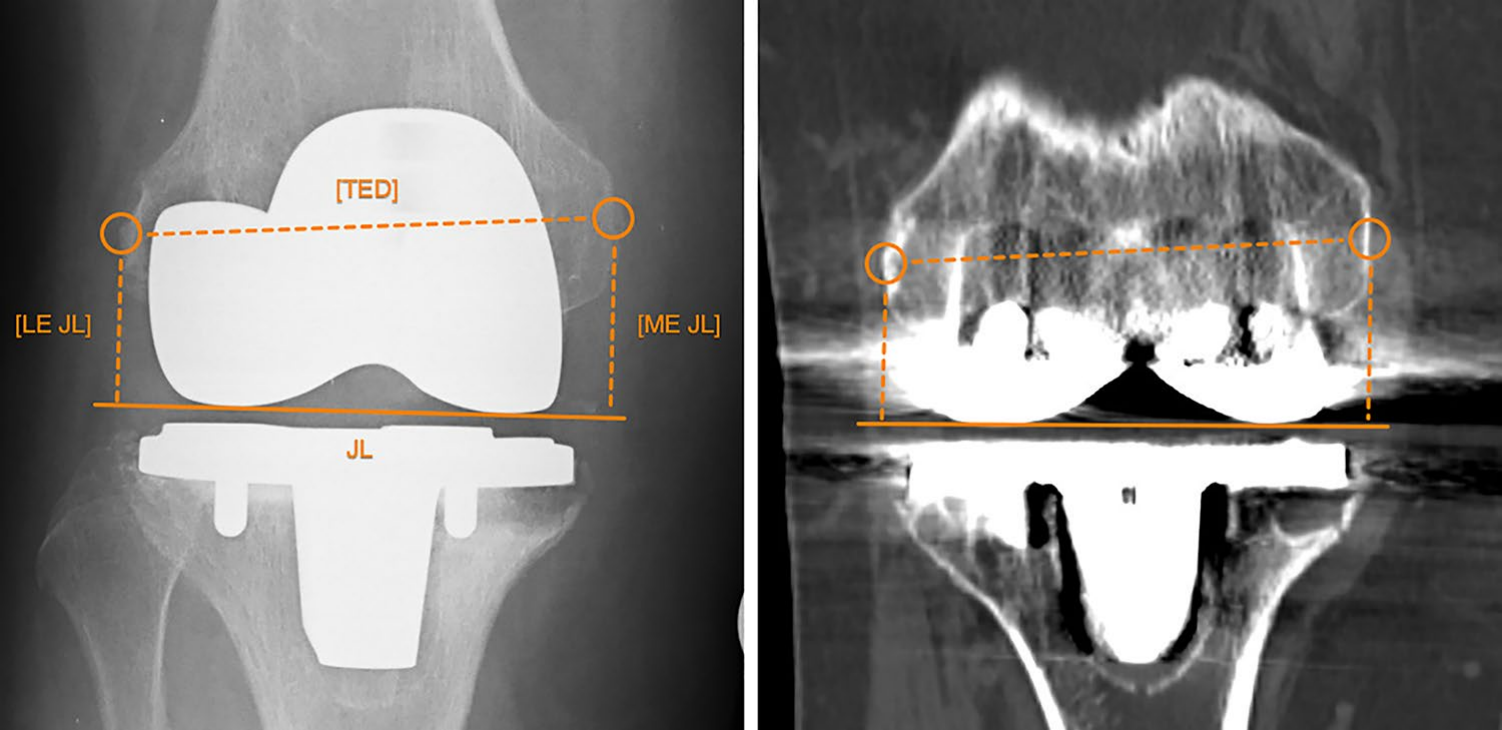
The epicondylar ratio. Measurement of the LER and MER in X-ray on the left and CT on the right. *TED* transepicondylar distance, *JL* joint line, *LE JL* distance between the lateral epicondyle and the joint line, *ME JL* distance between the medial epicondyle and the joint line

In both, X-ray of the knee and CT, the JL was aligned tangentially to condyles of the femoral component. The lateral epicondyle was defined as the most prominent distal lateral structure. On the medial side, the epicondylar sulcus was used. It was either represented by a small groove or crescent-shaped line (Fig. 1). To calculate the lateral ER (LER) and medial ER (MER) the perpendicular distance from the JL to the epicondyle was divided by the transepicondylar distance (TED) (Fig. 1).

### Statistics

All images were measured by two blinded observers and one observer measured twice with a time interval of more than two weeks. The intra- and interobserver agreement, as well as the correlation of the ER in CT and X-ray, was quantified with the intraclass correlation coefficient (ICC). The software R with the ‘irr’ package was used to calculate the ICC (agreement, two-sided). Values below 0.40 were interpreted as poor, between 0.40 and 0.59 as fair, between 0.60 and 0.74 as good and above 0.75 as excellent [5]. The 95% confidence intervals were calculated.

## Results

It was possible to measure the ER in all CT and X-ray images. Average values for the LER and MER are shown in Table 1.

**Table 1:** Average lateral and medial ER

	LER	MER
X-ray	0.32 (± 0.04)	0.34 (± 0.04)
CT	0.32 (± 0.04)	0.35 (± 0.04)

The ICC revealed a good to excellent intra- and interobserver agreement (Table 2).

**Table 2:** Intra- and Interobserver agreement (ICC) for lateral and medial ER in CT and X-ray

	Intraobserver Agreement	Interobserver Agreement
CT		
LER	0.93	0.74
MER	0.91	0.85
X-ray		
LER	0.88	0.81
MER	0.88	0.81

The correlation between the two imaging modalities was excellent with 0.81 for the LER and 0.79 for the MER.

## Discussion

The current study demonstrates comparable reliability of the measurement of the ER in X-ray and CT. Additionally, it reveals a correlation for the LER and MER between X-ray and CT.

The hypothesized higher reliability in CT compared to X-ray was not confirmed. When dealing with the ER in CT, a slice must be selected. Since the selected slice to be measured was up to the observer, small differences in the actual ER might be caused by the slice selection. Especially for the LER, the CT was inferior. Other differences in the agreement were interpreted as marginal.

Correlation between X-ray and CT is influenced by radiographic projection. Whereas CT allows measurement in different slices, X-ray images are always the result of radiographic projection, which depends on rotation and flexion [5, 7, 13]. This might reduce the validity and raise the reliability of the measurement. In case of extension lag, which leads to an a.p. X-ray of the knee in flexion, or rotational issues, the CT should be preferred. It remains to be discussed whether a 3D analysis with correct plain alignment is helpful to retrieve correct distances in CT.

The study of Servien et al. who first described the ER in a healthy patient collective in MRI, revealed an average LER of 0.28 and MER of 0.34 [15]. The present study analyzed malfunctioning TKA and therefore had different average LER and MER. General reference values of the ER in patients with TKA are not known.

The results are limited by the number of 107 cases. A lot of patients had to be excluded because of severe component dislocation or periprosthetic fracture. Apart from that the comparison of a 2D and 3D imaging modality limits the correlation. One of the observers already measured X-ray images in a previous study and noticed a learning curve being inherit to slight rotational and anatomical differences [8]. Therefore, both observers examined prior to the actual measurement not included images and discussed the handling of different radiographic projections.

The described ER can be used to determine the joint line in case of femoral bone loss with existing epicondyles in rTKA. The preoperatively calculated LER and MER would be multiplied with the intraoperatively caliper-measured TED to obtain the distance from the epicondyle to the JL lateral and medial. Nevertheless, one should bear in mind that epicondyles can be palpated intraoperatively with an accuracy of about ± 2–5 mm [16]. In cases with extensive femoral bone loss, tibia-based methods like the Figgie distance are still relevant. Since there is no clearly superior technique, the ER can be used in conjunction with other methods like the Figgie distance.

## Conclusion

The measurement of the ER in X-ray and CT is reliable and results of the two imaging modalities correlate. Prior to rTKA, a sole determination of the ER in X-ray is sufficient.
